# Supplementary material for: Cell cycle criticality as a mechanism for robust cell population control
Source: Mol Syst Biol. 2025 Nov 6;22(2):4. doi: 10.1038/s44320-025-00164-8 (PMC12864836; doi:10.1038/s44320-025-00164-8)
Supplement: Supplementary file 2 — Expanded View Figures [file 44320_2025_164_MOESM2_ESM.pdf]

Expanded View Figures

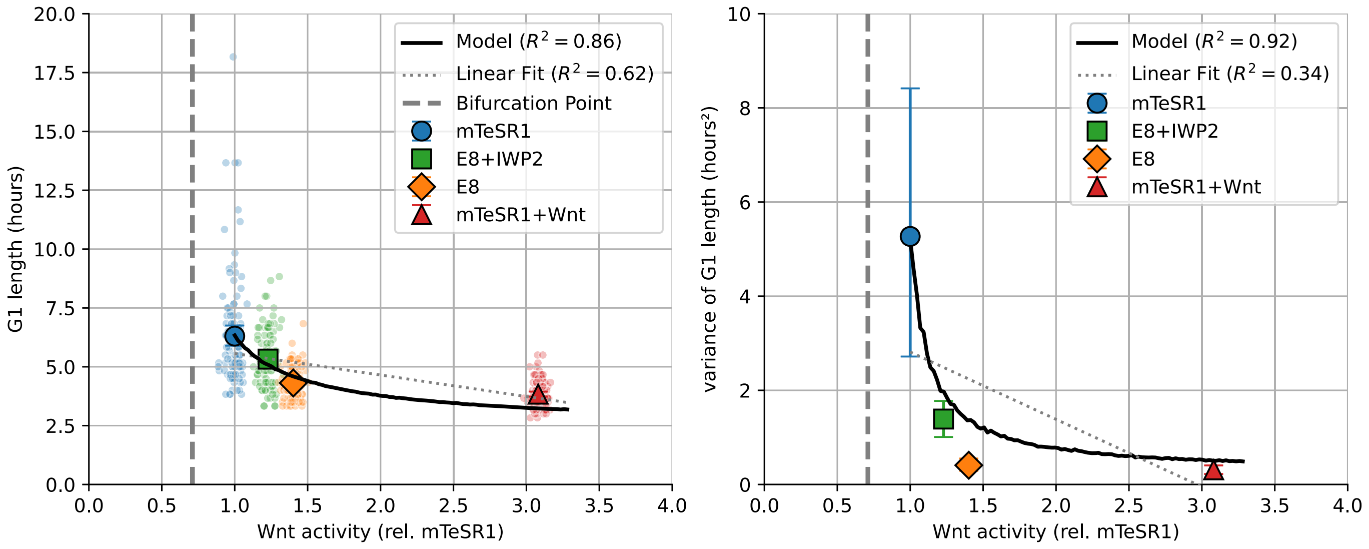

Figure EV1. Quantitative comparison of G1 lengthening models.

Comparison of experimental data ($n = 114$ for mTeSR1, $n = 105$ for E8+IWP2, $n = 104$ for mTeSR1+Wnt, and $n = 112$ for E8) from Jang et al (2019) with model predictions for the mean (left) and variance (right) of G1 length in hESCs under different Wnt signalling conditions. The model, based on a noisy saddle-node bifurcation (solid black line), captures the dependency of both the mean ($R^2 = 0.86$) and the variance ($R^2 = 0.92$) of G1 length on Wnt activity, outperforming linear regressions (dotted grey line, $R^2 = 0.62$ and $R^2 = 0.34$, respectively). Experimental data points show bootstrapped means (centre of error bars) with 95% confidence intervals. The dashed vertical line indicates the bifurcation point.

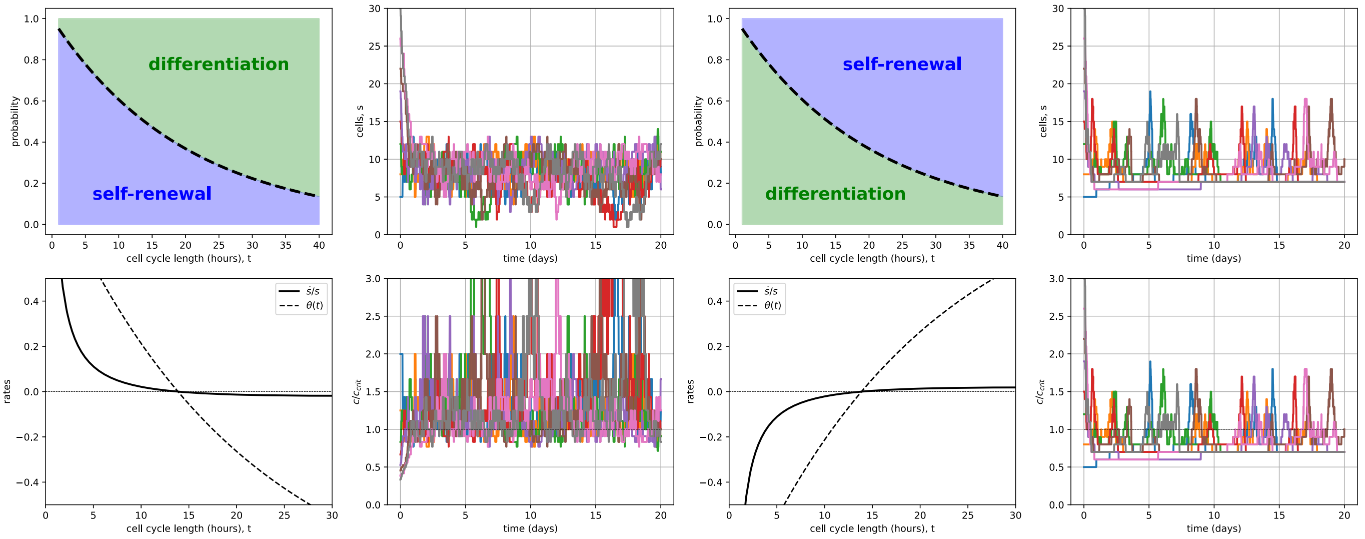

Figure EV2. Robust population control in small stem cell niches.

To test the robustness of our proposed control mechanism, we simulated the population dynamics for both the RD (left) and DR (right) topologies in a small stem cell niche with a target set-point of $N = 10$ cells. The simulations show that even in the presence of significant demographic fluctuations inherent to small populations, the negative feedback loop effectively maintains the population size around the set-point. In both topologies, the system consistently self-tunes to the vicinity of the critical point (bottom right panels), demonstrating that the control mechanism is robust and does not require a large population size to function.

 © The Author(s)
